# Types of Transnasal Endoscopic Nasopharyngectomy for Recurrent Nasopharyngeal Carcinoma: Shanghai EENT Hospital Experience

**DOI:** 10.3389/fonc.2020.555862

**Published:** 2021-01-14

**Authors:** Quan Liu, Xicai Sun, Han Li, Jiaying Zhou, Yurong Gu, Weidong Zhao, Houyong Li, Hongmeng Yu, Dehui Wang

**Affiliations:** ^1^ Department of Otolaryngology, Eye, Ear, Nose and Throat Hospital, Shanghai Medical College of Fudan University, Shanghai, China; ^2^ Research Units of New Technologies of Endoscopic Surgery in Skull Base Tumor, Chinese Academy of Medical Sciences, Beijing, China

**Keywords:** nasopharyngeal carcinoma, local recurrence, endoscopy, endoscopic skull base surgery, nasopharyngectomy

## Abstract

**Background:**

Transnasal endoscopic nasopharyngectomy (TEN) has become increasingly used for recurrent nasopharyngeal carcinoma (rNPC); however, there is no report on the definitive resectable contour for TEN according to the latest staging system for nasopharyngeal carcinoma. The aim of this study was to establish the types of TEN for rNPC.

**Materials and Methods:**

A total of 101 rNPC patients underwent TEN from January 2016 to April 2019 at the authors’ institution. TEN was categorized into four types, which included type I (n=40) with resection of the nasopharynx and sinuses; type II (n=10) with lateral extension to the parapharyngeal space; type III (n=40) with lateral extension to the floor of the middle cranial fossa and the infratemporal fossa and superior extension to the orbital apex and the cavernous sinus back to the prevertebral region; and type IV (n=11) with the resection of the involved internal carotid artery following type III. The 2-year overall survival rate (OS) and local recurrence-free survival rate (LRFS) were assessed.

**Results:**

The median time of follow-up was 20 months. Twenty-five patients reoccurred. Nineteen patients died. Independent predictors of outcome on multivariate analysis were recurrent T stage (P = 0.039), types of TEN (P = 0.002) and surgical margin (P = 0.003). The 2-year OS and LRFS was 76.2% and 53.6%, respectively.

**Conclusions:**

The result of TEN in the treatment of rNPC is promising. The types of TEN will provide effective guideline for surgical treatment of rNPC.

## Introduction

Salvage surgical resection has been considered as the first line of treatment of residual or recurrent nasopharyngeal carcinoma (rNPC) at the primary site (1). Due to the deep location of the nasopharynx, surgery for local rNPC has conventionally been performed through open approaches, including transpalatal, transmandibular, maxillary swing, or transinfratemporal approaches, which are often associated with considerable morbidity (2). With advances in expanded endoscopic transnasal surgery, emerging evidence has demonstrated the encouraging results of endoscopic nasopharyngectomy for local rNPC that occurs at the primary site as well as advanced rNPC (3–5).

For endoscopic transnasal nasopharyngectomy, P. Castelnuovo (6) first reported the types of the nasopharyngeal endoscopic resection (NER), which were categorized into three different types. Both type 1 and type 2 NER essentially target rNPC located in the middle line of the skull base, including the nasal sinus and the nasopharyngx. Type 3 NER extends laterally to the parapharyngeal space and the ipsilateral cartilagineous portion of the eustachian tube; however, the types of NER are limited for use in terms of advanced rNPC, especially for internal carotid artery involvement. Even with the update of the staging system for rNPC, to the authors’ best knowledge, there is still no type of surgical nasopharyngectomy according to the tumor staging of rNPC.

Herein, the types of transnasal endoscopic nasopharyngectomy (TEN) are introduced based on the latest staging system for nasopharyngeal carcinoma (AJCC, 2010), and its application in the management of advanced rNPC is evaluated.

## Materials and Methods

### Patients

A total of 101 rNPC patients underwent TEN at the authors’ institution from January 2016 to April 2019. There were 67 men and 34 women. The median age was 51 years. According to the American Joint Committee on Cancer (AJCC) classification, 8th edition, 31, 10, 31, and 29 patients were classified as rT1, rT2, rT3, and rT4, respectively. Among these patients, regional lymph node metastases were present in 18 patients. There was no patient with distant metastases. Type I of TNE was performed in 40 patients, type II in 10 patients, type III in 40 patients, and type IV in 11 patients. In the first year after surgery, there was a follow-up on patients every three months with an endoscopic examination of the nasopharynx at each visit. Magnetic resonance imaging (MRI) of the nasopharynx was performed every six months. These examinations were spaced out progressively during the subsequent years after surgery. This study was approved by the Research and Ethics Committee of EENT Hospital affiliated with Fudan University in China.

### Anatomical Establishment of TEN

Transnasal endoscopic nasopharyngectomy was categorized into four types based on the stepwise exposed anatomical structures and clinical T staging for rNPC.

#### Type I TEN

**Figure 1:**
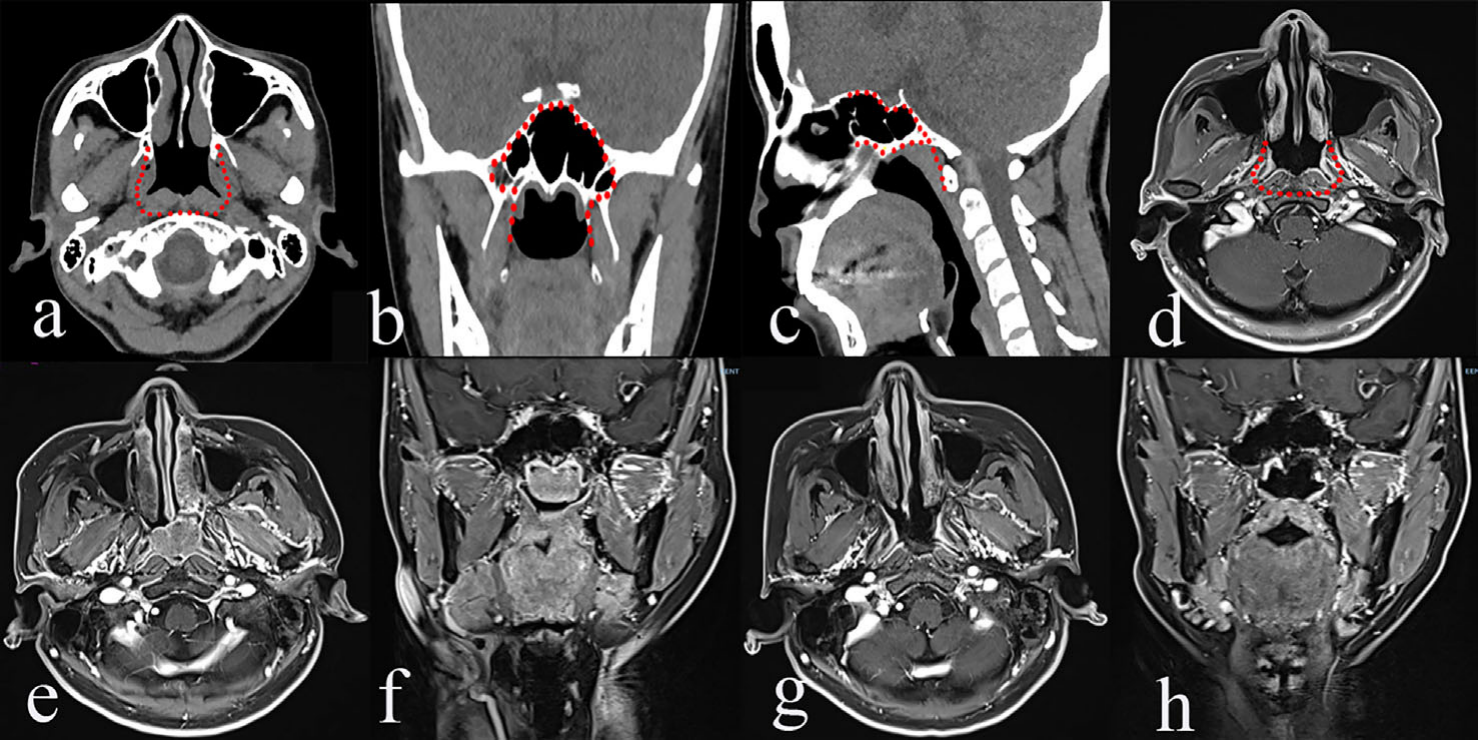
The greatest extent of resection in Type I transnasal endoscopic nasopharyngectomy (TEN) [red dot line in **a–d**], pre-operative magnetic resonance imaging (MRI) of rT1 nasopharyngeal carcinoma (NPC) **(e, f)**, and post-operative MRI **(g, h)**.

It is designed to treat rNPC that occurs in the midline of the nasopharynx and skull base. The greatest extent of resection includes medial paraclival internal carotid artery (ICA), the cartilaginous Eustachian tube and the medial pterygoid plate laterally, the longus capitis muscle posteriorly, the sphenoid planum superiorly, the nasal cavity and ethmoid sinus anteriorly, and the oropharynx inferiorly. Herein, the contour of type I TEN targets rT1 and rT3 rNPC that occurs in the midline of the skull base (**Figure 1**). In our study, a total of 40 patients underwent type I TEN, including 31 rT1 and 9 rT3 with involvement of the midline of the skull base.

#### Surgical Procedure

If a tumor is confined within the posterior nasopharyngeal wall, the endoscopic bi-nostril approach is performed. Initially, the endoscopic transnasal bilateral inferior turbinate outfracture and the partial posterior septectomy are routinely performed to optimize visualization of the nasopharynx. The extent of resection is pharyngobasilar fascia posteriorly, basisphenoid superiorly, and torus tubarius laterally. A resection of the tumor starts from the basisphenoid downwards along prevertebral muscles. The tumor should be resected en-bloc. In terms of a sphenoid sinus involvement of the tumor, the ipsilateral partial middle turbinate is resected, followed by bilateral sphenoidotomies by drilling out the intersphenoidal septum, sphenoid rostrum, and the floor of the sphenoid sinus. The lateral margin is extended to the medial pterygoid plate.

#### Type II TEN

The resection is extended laterally to the upper parapharyngeal space (PPS) and the cartilagineous portion of the Eustachian tube. The type II procedure is bounded by the paraclival ICA, the lacerum ICA, lateral pterygoid plate laterally, and the pharyngobasilar fascia posteriorly. The anterior boundary is the pterygoid base. The superior and inferior boundaries are similar to type I TEN.

#### Surgical Procedure

**Figure 2:**
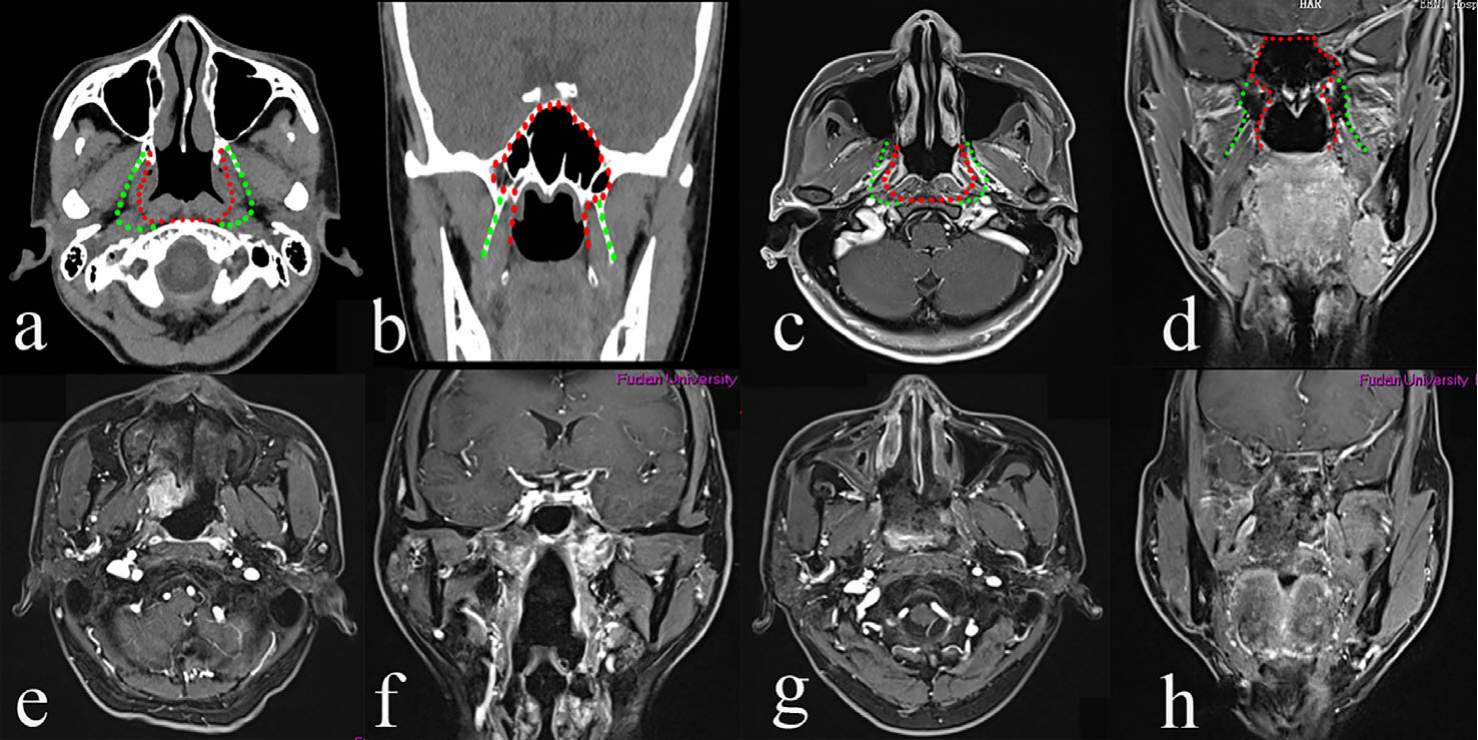
The greatest extent of resection in Type II transnasal endoscopic nasopharyngectomy (TEN) [green dot line in **(a–d)**], pre-operative magnetic resonance imaging (MRI) of rT2 nasopharyngeal carcinoma (NPC) **(e, f)**, and post-operative MRI **(g, h)**.

Initially, the uncinectomy and the enlargement of the maxillary ostium are performed, followed by an anteroposterior ethmoidectomy to expose the medial wall of the orbit. The ipsilateral middle turbinate is removed. The transethmoidal sphenoidotomy is performed. Landmarks of the paraclival carotid artery, optic nerve, sellar floor, and anterior wall of the cavernous sinus are identified. The sphenopalatine artery (SPA) is exposed and cauterized by the removal of the crista ethmoidalis, followed by the drilling out of the perpendicular plate of the palatine bone. The descending palatine artery and the greater palatine nerve are cauterized. The pterygopalatine fossa is exposed by the removal of the posterior wall of the maxillary sinus using Kerrison’s rongeurs. A lateral displace of the pterygopalatine fossa is performed to identify the pterygoid base and to expose and cut the palato-vaginal artery and vidian canal nerve. The foramen rotundum is located superior-lateral of the vidian nerve. Anteroposterior drilling along the vidian nerve is performed to expose the anterior genu of the ICA. The pterygoid base is drilled out to expose the pterygoid plates and the medial pterygoid muscle. The medial pterygoid plate is drilled out to expose the tensor veli palatine muscle. At this stage, the pre-styloid fat tissue of the upper PPS comes into view between the tensor veli palatine muscle (TVPM) and the medial pterygoid muscle (MPM). The TVPM is cut to expose the cartilagineous portion of the Eustachian tube and the levator veli palatini muscle, which should be resected to expose the retro-styloid compartment of the upper PPS. Tensor-vascular-styloid (TVS) fascia is just anterior to the parapharyngeal ICA. Thus, the type II TEN was designed for the rT2 rNPC (**Figure 2**).

#### Type III TEN

The procedure is extended laterally to the infratemporal fossa, the bone portion of the Eustachian tube, orbital and superior orbital fissure, the floor of the middle cranial fossa, cavernous sinus, pericranial nerve, and cervical spine. The medial bound is the lateral pterygoid plate, foramina ovale, and sphenoid spine. The lateral boundary is the bone portion of the Eustachian tube, lateral margin of the lateral pterygoid muscle (LPM) and the deep lobe of parotid gland. The anterior boundary is the posterior wall of the maxillary sinus. The posterior bound is the cervical spine.

#### Surgical Procedure

**Figure 3:**
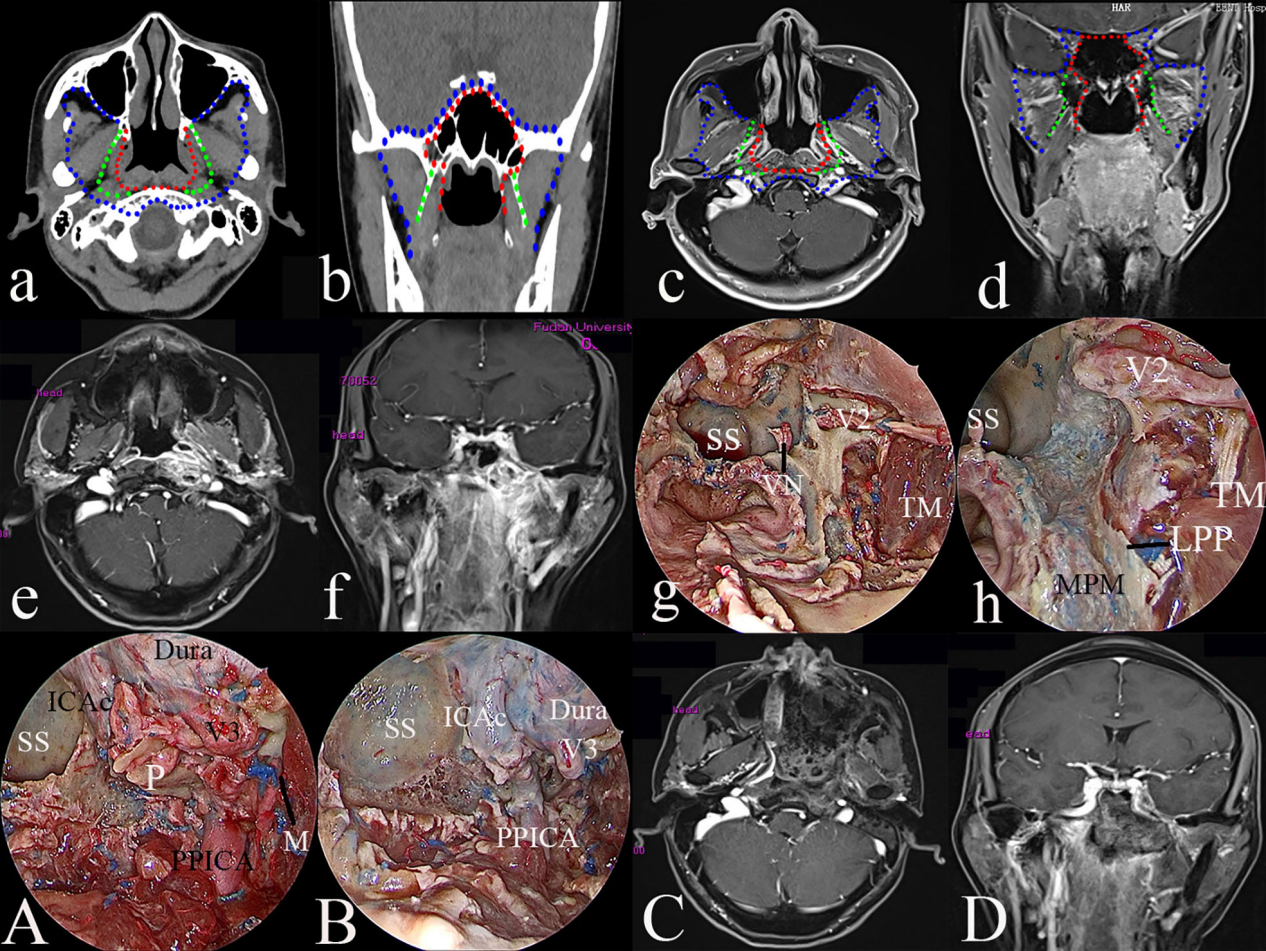
The greatest extent of resection in Type III transnasal endoscopic nasopharyngectomy (blue dot line in **a–d**), pre-operative magnetic resonance imaging (MRI) of rT3 nasopharyngeal carcinoma (NPC) **(e, f)**; exposure of the key landmarks in Type III TEN **(g, h, A, B)**; and post-operative MRI **(C, D)**. ICAc, clival internal carotid artery; LPP, the lateral pterygoid plate; MPM, medial pterygoid muscle; M, middle meningeal artery; P, petrous bone; PPICA, parapharyngeal internal carotid artery; SS, sphenoid sinus; TM, temporal muscle; VN, vidian nerve; V2, maxillary nerve; V3, mandibular nerve.

To obtain better manipulation for the workplace and exposure, a combined transnasal endoscopic transpterygoidal and anterior transmaxillary approach is performed. After the establishment of the surgical field of the type II TEN, the paraclival, foramen lacerum, and petrous segments of ICA are identified. The foramen rotundum and maxillary nerve are dissected superior-lateral to expose the superior orbital fissure and the maxillary strut. The medial bone of the middle cranial fossa is drilled out posteriorly along the maxillary nerve to access the Meckel’s cave. The anterior wall of the maxillary sinus is drilled out to further laterally expose the infratemporal fossa as needed. The maxillary artery and its branches are cauterized to remove the vascular net of the infratemporal fossa to identify the lateral pterygoid muscle and temporal muscle. Detachment of the lateral pterygoid muscle from the lateral pterygoid plate and spine of the temporal bone is performed, allowing for approaching the floor of the middle cranial fossa, foramina ovale, mandibular nerve, and its major branches. The lateral pterygoid plate and lingual process of the sphenoid are drilled out to expose the petrous clivus region. The petrous bone is drilled out as needed to expose the horizontal segment of the ICA. A Doppler ultrasound is useful for identifying the position of the ICA. If the tumor has invaded the floor of the middle cranial skull base, the greater wing of the sphenoid should be drilled out. Type III TEN aims to resect the tumor of rT3 (paramedial) and rT4 (extracranial) rNPC (**Figure 3**). The type III TEN was performed in 40 patients, including 22 rT3 and 18 rT4 rNPC without involvement of the ICA.

#### Type IV TEN

**Figure 4:**
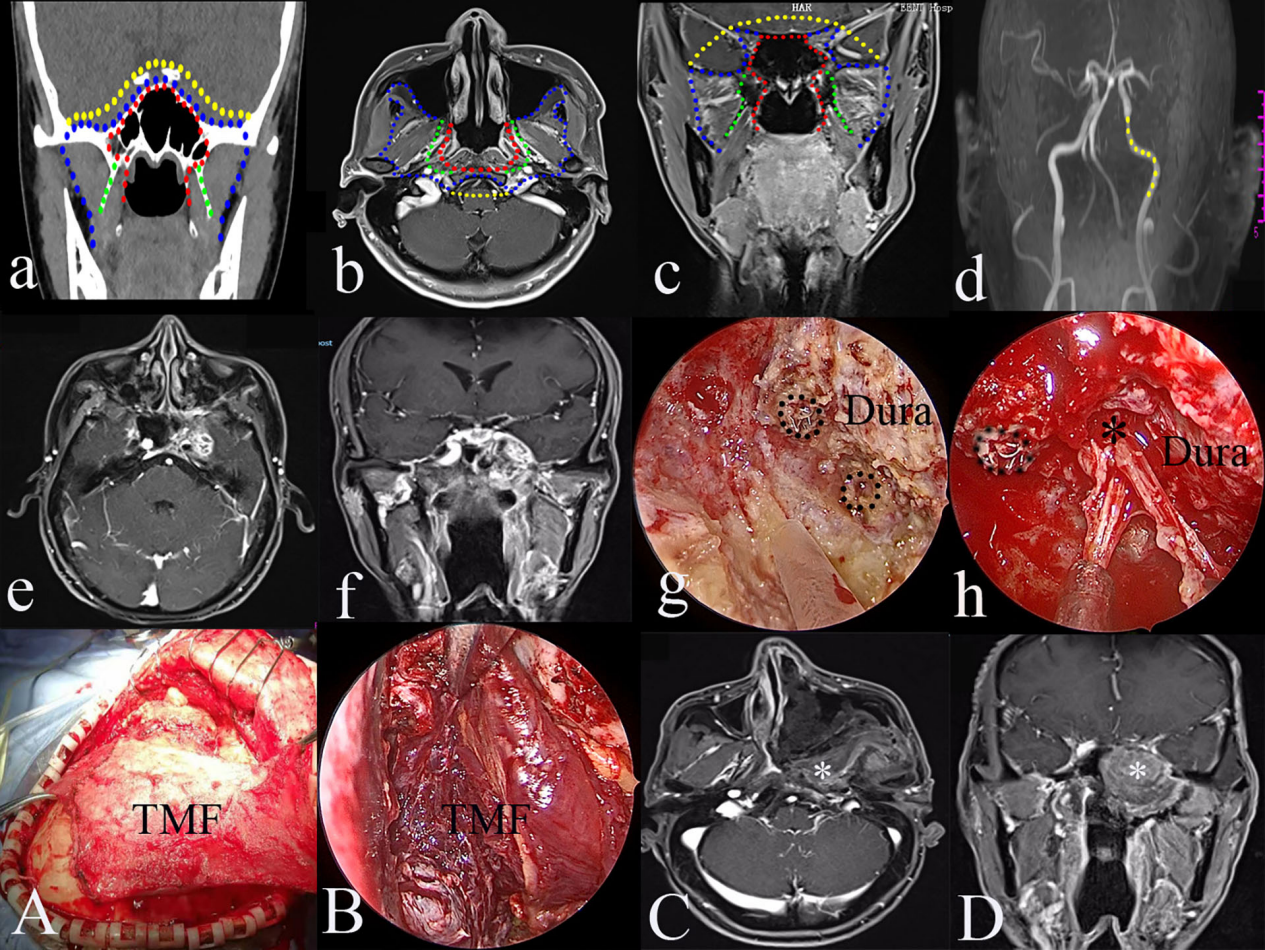
The greatest extent of resection in Type IV transnasal endoscopic nasopharyngectomy (yellow dot line in **a–d**); yellow dot line indicating the resectable internal carotid artery (ICA) **(d)**; pre-operative magnetic resonance imaging (MRI) of rT4 nasopharyngeal carcinoma (NPC) **(e)**; pre-operative MRA of left occluded ICA **(f)**; resection of the involved ICA (black dot circle) **(g)**; resection of the tumor in the Meckel’s cave **(h)**, black dot circle indicating resected ICA; the reconstruction of the skull defects using TMF **(A, B)**; post-operative MRI indicating the total resection of the tumor **(C, D)**. black *, Meckel’s cave; white *, temporalis muscle flap.

Type IV targets the resection of the involved ICA and rT4 (intracranial) rNPC following type III. The resected compartments of the ICA can be from the paraclival to the parapharyngeal segments (**Figure 4D**).

#### Surgical Procedure

The procedure targets rNPC patients with an intracranial ICA invasion. The greatest exposure of the type III procedure provides a better working place to manipulate extracranial ICA. If there is an ICA involvement of the tumor, curative surgery necessitates the resection of the tumor and ICA. Pre-operative balloon occlusion test is mandatory to make the decision regarding the ICA (**Figure 4**).

### Statistical Analysis

The statistical package for the social sciences version 17 (IBM SPSS Statistics 17) was utilized for the statistical analysis. The Kaplan–Meier analysis was used to calculate the OS and LRFS. The OS and LRFS were censored at the date of the last follow-up. All survival rates were also censored at two years if patients were still alive. Patients lost to follow-up are considered to be death. The Cox-Regression was performed to analyze the potential prognostic factors. A value of p<0.05 was considered significant.

## Results

### Clinical Application of the TEN Into the Management of rNPC

None of the patients had severe complications during the operations. The median operation time was 203 min (range, 50–611 min). The nasoseptal flap was used for the reconstruction of skull base defects in 58 patients, turbinate mucosa in 19 patients, and the temporalis muscle flap in six patients.

The preoperative contrast MRI showed that 11 patients (10.9%) had radiological evidence of ICA involvement. To achieve radical extirpation, the involved ICA was occluded after the balloon occlusion test except for one patient, who received an extracranial–intracranial vascular bypass as a result of the failure of the balloon occlusion test. For these patients, the type III TEN was first performed to resect the tumor, and then the involved ICA was resected using the type IV TEN. At the end of the surgery, the temporalis muscle flaps were used to completely cover the exposed skull base defects in six patients.

Fourteen patients received chemotherapy, and five patients had adjuvant radiotherapy. The median time of follow-up was 20 months (range 5–44 months). Twenty-five patients reoccurred after surgery. Nineteen patients died: one of local disease, two of distant metastasis, six of massive bleeding, one of other disease, three of necrosis, two of unknown reasons and four patients lost to follow-up. There were total of 11 patients with massive bleeding after surgery (ranging 3 to 31 months). Six patients died of massive bleeding, including four patients of type III TEN with nasoseptal flap reconstruction and two patients of the type I TEN. The massive bleeding occurred in the remaining five patients were successfully controlled (**Table 1**).

**Table 1:** Characteristics of patients and univariate analysis of prognostic factors for overall survival (OS) and local recurrence-free survival rate (LRFS).

Variables	No. of the patients (%)	P value	
		2-year OS	2-year LRFS
Age		0.848	0.483
< 50	46(45.5)		
≥50	55(54.5)		
Sex		0.164	0.416
Male	67(66.3)		
Female	34(33.7)		
Recurrent T staging		0.106	0.039
1	31(30.7)		
2	10(9.9)		
3	31(30.7)		
4	29(28.7)		
Types of TEN		0.275	0.002
I	40(39.6)		
II	10(9.9)		
III	40(39.6)		
IV	11(10.9)		
Reconstruction		0.219	0.234
None	18(17.8)		
Turbinate mucosa	58(57.4)		
Nasalseptal flap	19(18.8)		
TMF	6(5.9)		
Post-operative radiotherapy		0.253	0.075
Yes	14(13.9)		
No	83(82.2)		
Post-operative chemotherapy		0.207	0.097
Yes	5(5)		
No	92(91.1)		
Margin		0.375	0.003
Positive	16(15.8)		
Negative	85(84.2)		
Massive bleeding		0.781	0.113
Yes	11(10.9)		
No	86(85.1)		

TEN, transnasal endoscopic nasopharyngectomy; TMF, temporalis muscle flap; OS, overall survival; LRFS, local recurrence-free survival.

**Figure 5:**
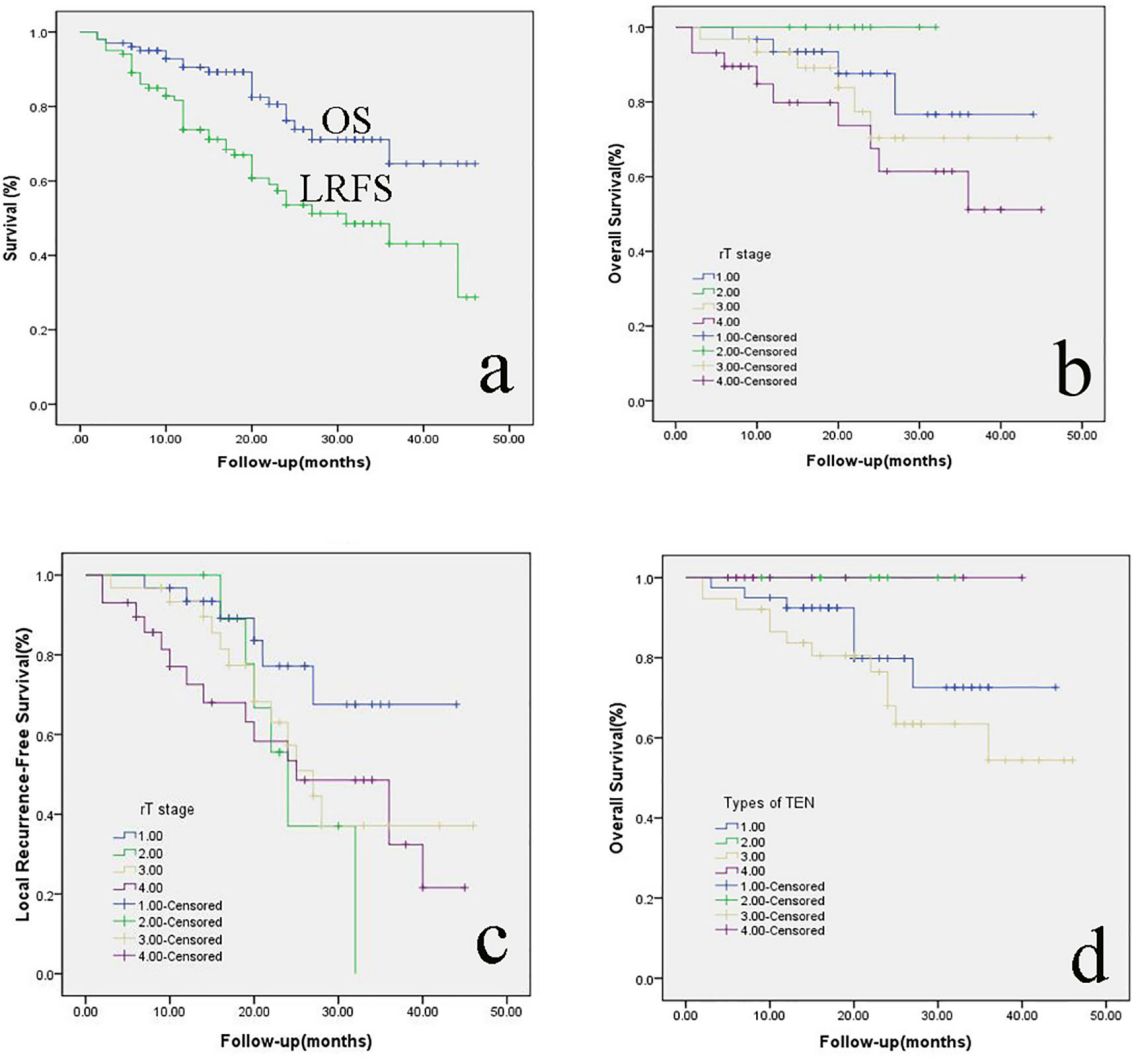
**(A)** the 2-year OS and LRFS of rNPC patients; the 2-year OS **(B)** and LRFS **(C)** of rNPC with different T stages; **(D)**: the 2-year OS of rNPC with different types of TEN. OS, overall survival; LRFS, Local recurrence-free survival; rNPC, recurrent nasopharyngeal carcinoma; TEN, transnasal endoscopic nasopharyngectomy.

The 2-year OS was 76.2% (**Figure 5A**). The 2-year OS for rT1, rT2, rT3, and rT4 was 87.6%, 100%, 70.4%, and 67.5%, respectively (**Figure 5B**). The 2-year LRFS was 53.6% (**Figure 5A**). The 2-year LRFS for rT1, rT2, rT3, and rT4 was 77.2%, 37%, 57.3%, and 53.4%, respectively (**Figure 5C**). In terms of the types of TEN, the 2-year OS for type I, type II, type III, and type IV was 79.8%, 100%, 68.0%, and 100%, respectively (**Figure 5D**). Cox-regression analysis showed that recurrent T stage (P = 0.039), types of TEN (P = 0.002) and surgical margin (P = 0.003) could be used as an independent prognostic factor for OS. There were no significant associations between other prognostic factors such as age, sex, primary T staging and radiotherapy after surgery.

## Discussion

Nasopharyngeal carcinoma (NPC) remains endemic in southern China, with a peak annual incidence approaching 30 per 100,000 persons (7). Based on the fact that the majority of primary NPC are undifferentiated or nonkeratinizing with the characteristic of being radiotherapy sensitive, the mainstay treatment for the primary NPC is radiotherapy associated with/without chemotherapy. With advances in radiotherapy planning and delivery techniques, such as intensity-modulated radiotherapy (IMRT) and the use of chemoradiation, the 5-year local control rate of 76% to 91% can be achieved (8).

After the initial radical radiotherapy with or without chemotherapy, up to 15% of primary NPC will recur, which mostly occurs within the first to the third year after treatment (9). Salvage treatment for rNPC has remained challenging, and the current options include surgery, re-irradiation, or chemotherapy (10, 11). Emerging evidence demonstrates that re-irradiation is associated with severe late complications and results in decreased quality of life. A massive hemorrhage of the nasopharynx is the major severe late complication and also the leading cause of death (9, 12).

Salvage surgical resection for the rNPC can avoid the complications associated with re-irradiation. More recently, endoscopic transnasal nasopharyngectomy for rNPC is preferred due to the advantages of high magnification and avoiding the morbidities inherent with open procedures. Increasing data support minimal morbidity and similar survival outcomes to open approaches or re-radiation (13).

Castelnuovo and colleagues first reported the types of the endoscopic transnasal nasopharyngectomy (6). With advances in the endoscopic surgical technique and instruments, endoscopic transnasal approaches to the skull base are an increasing focus, especially in the treatments of the tumors involving the ICA (14). For tumors with carotid artery involvement, removing the tumor including the ICA could be named type 4 NER as a complement of the Castelnuovo’NER classification (5). Based on our previous report, the modified classification system proposed categorizes the endoscopic nasopharyngectomy into four types according to the anatomic structures progressively removed.

### Type I TEN

It focuses on rNPC that occurs in the posterior nasopharynx wall, sphenoid sinus, nasal cavity, and midline skull base. Thus, both rT1 and rT3 (confined within the midline skull base) rNPC should be treated using type I TEN. The data of this study revealed that the 2-year OS for rT1 was 87.6% similar to the 2-year OS of 79.8% of type I TEN. Thus, in terms of rNPC involving the middle line of the nasopharynx and the skull base, type I TEN can be an alternative surgical approach.

rNPC usually invades laterally to the PPS. Surgical management of lesions in the PPS is challenging due to adjacent critical neurovascular structures. An anatomical study of the PPS using an endoscopic transnasal transmaxillary transpterygoid approach was reported in 2010 by Taniguchi and Kohmura (15), indicating that the approach would be restricted to benign tumors and inflammatory processes or limited-sized malignant tumors. From an anatomical point of view, the endoscopic transnasal with/without transoral approach to the PPS is minimally invasive (16). In this study, type II was performed on 10 patients with rNPC invading the PPS. Our results demonstrated that 2-year OS and LRFS for rT2 was 100% and 37%, respectively. Six of ten patients with rT2 recurred during the follow up and the mean time of recurrence was 12 months after the surgery. The complex of the PPS and residual tumor involving the ICA, in our opinion, may contribute to the unsatisfying LRFS. In the type II surgery, locating the ICA was critical to avoid severe complications. The root of the pterygoid process and medial pterygoid plate were drilled to expose the PPS. The TVPM and the tensor-vascular-styloid fascia were used to locate the parapharyngeal ICA (17). The pharyngobasilar fascia was used to indicate the lacerum segment of the ICA. Furthermore, the preoperative MRA is helpful in identifying the variation of the parapharyngeal ICA (18, 19).

Type III surgery was employed to treat advanced rNPC. During the surgery, the lateral petroclival region, infratemporal fossa, the floor of the middle cranial fossa, superior orbital fissure, cavernous sinus, and prevertebral region should be effectively exposed. Herein, rT3 and rT4 rNPC were the targets of type III TEN. To perform type III TEN, a combined endoscopic transnasal and anterior transmaxillary approach was suggested, which offered sufficiently wide exposure to ensure complete removal using bimanual techniques and facilitated the control of bleeding, especially bleeding from the ICA. Navigation or Doppler should be used to identify the ICA. The great wing of the sphenoid could be drilled away as needed. The dura can be resected if there is tumor involvement. The negative surgical margin should be confirmed using frozen sections. At the end of the surgery, reconstruction is recommended using the temporalis muscle flap to cover the exposed extracranial internal carotid artery to prevent serious complications, such as massive hemorrhage, infection, and necrosis.

ICA involvement was common in patients with the advanced stage of rNPC. Conventionally, palliative treatment with chemotherapy has been used for these patients. A catastrophic massive hemorrhage from the ICA was the common reason of death. In this study, all the patients underwent preoperative MRI and MRA. There were 11 patients with ICA involvement. For a radical resection of the tumor, the involved ICA was blocked after the balloon occlusion test or bypass method. The aim of the type IV TEN is to resect the involved ICA after the type III procedure. There were 11 patients with ICA involvement undergoing type IV TEN in this study. To date, these patients have recovered well except one, who experienced a recurrence five months after surgery and received chemotherapy.

In terms of the skull base reconstruction, the temporalis muscle flap has the ability to meet the needs of type III and type IV TEN. The temporalis muscle flap has become extensively used in tissue reconstruction of the skull base, maxillofacial, oral cavity, and oropharyngeal defects after open oncological procedures since its first use in reconstructing the orbit after an exenteration of the eye by Golovine in 1898 (20, 21). In this study, the temporalis muscle flap was used for six patients, including two type III and four type IV. Six patients died from massive bleeding, and four patients underwent type III TEN with the nasalseptal flap reconstruction. Massive bleeding did not occur in patients who underwent type III TEN with temporalis muscle flap reconstruction. When tumors invade the parapharyngeal space or the petrous segment of the internal carotid artery, the medial and lateral pterygoid muscles and part of the pharyngeal basilar fascia must be removed. Therefore, the petrous and parapharyngeal segments of the internal carotid artery may be exposed during the operation, and some patients may die from carotid artery blowout after surgery (22). Thus, the temporalis muscle flap reconstruction should be performed for type III and type IV TEN to prevent carotid rupture and osteoradionecrosis of the skull base.

The main limitation of our study is that the duration of the follow up is short and that the study was performed by retrospective analyses. Herein, future prospective studies and long-term follow up are needed to confirm the outcomes of the types of TEN in the treatment of rNPC. Despite its limitation, we believe that the application of the types of TEN helps to improve the treatment of the rNPC.

## Conclusions

The types of TEN provide a more detailed contour for the endoscopic surgical treatment of rNPC. TEN is effective and successful in treating different T stages of rNPC. The preliminary results indicate that types of TEN may be an effective guideline for the endoscopic surgical treatment of rNPC. Although the preliminary results are encouraging, more clinical experiences and long-term follow-up are still needed to validate the results.

## Data Availability Statement

The original contributions presented in the study are included in the article/supplementary material. Further inquiries can be directed to the corresponding authors.

## Ethics Statement

This study was approved by the Research and Ethics Committee of EENT Hospital affiliated with Fudan University in China.

## Author Contributions

HY and DW conceived, designed and supervised the study. QL, XS, HL, and JZ wrote the manuscript. QL, JZ, YG, WZ, and HYL collected and analyzed the data. All authors contributed to the article and approved the submitted version.

## Funding

This work was supported by the New Technologies of Endoscopic Surgery in Skull Base Tumor: CAMS Innovation Fund for Medical Sciences (CIFMS) (2019-I2M-5-003), Chinese Academy of Medical Sciences, Shanghai Hospital Development Center (SHDC12018118, SHDC2020CR2005A), and Shanghai Science and Technology Committee (19411950600).

## Conflict of Interest

The authors declare that the research was conducted in the absence of any commercial or financial relationships that could be construed as a potential conflict of interest.
